# Association Between Psychological Distress and Glycaemic Control Among Adults with Diabetes Mellitus in Saudi Arabia

**DOI:** 10.3390/healthcare14182978

**Published:** 2026-09-11

**Authors:** Fahad Abdulaziz Alrashed, Tauseef Ahmad, Syed Irfan Karim, Jennesse John

**Affiliations:** 1 Department of Medical Education, College of Medicine, King Saud University, P.O. Box 2925, Riyadh 11472, Saudi Arabia; tahmad@ksu.edu.sa (T.A.); jaresseril@ksu.edu.sa (J.J.); 2 Department of Family and Community Medicine, College of Medicine, King Saud University, P.O. Box 2925, Riyadh 11472, Saudi Arabia; skarim@ksu.edu.sa

**Keywords:** glycaemic control, psychological distress, chronic metabolic, diabetes, medical education, health-care

## Abstract

**Background:** Diabetes mellitus is a chronic metabolic disorder often accompanied by psychological distress, including stress, anxiety, and depression. Such emotional symptoms can influence self-care behaviours and metabolic control. In Saudi Arabia, cultural and religious practices may mitigate the effects of psychological distress on glycaemic regulation. Limited evidence exists regarding the simultaneous assessment of depression, anxiety, and stress in relation to objective glycaemic indicators such as HbA1c among adults with diabetes in Saudi Arabia. This study aimed to determine the association between psychological distress and glycaemic control among adults with diabetes, while exploring the influence of demographic and biochemical factors. **Methods:** A cross-sectional study was conducted from 21 January 2026 to 19 April 2026 among 546 adults diagnosed with diabetes in multiple healthcare settings in Riyadh, Saudi Arabia. Psychological distress was assessed using the DASS-21 questionnaire, while glycaemic control was measured using HbA1c, categorised as controlled (<7%) or uncontrolled (≥7%). Demographic, anthropometric, and biochemical parameters including BMI, blood pressure, and lipid profile were collected from medical records and standardized assessments. Associations were examined using chi-square tests and multiple linear regression analysis was performed including psychological distress scores, BMI, blood pressure measurements, and lipid profile parameters to identify factors associated with HbA1c variation. **Results:** Psychological distress was prevalent; however, stress (6.2–10.7%), anxiety, and depression were significantly associated with variation in HbA1c levels. In contrast, nationality, total cholesterol, LDL, triglycerides, and systolic blood pressure were significantly associated with HbA1c. Multiple regression showed that stress (B = 0.09, *p* = 0.015), systolic blood pressure (B = 0.146, *p* = 0.006), and total cholesterol (B = 0.229, *p* < 0.001) were independent predictors, collectively explaining 14.8% of HbA1c variance. Multiple regression analysis identified stress, systolic blood pressure, and total cholesterol as significant factors associated with variation in HbA1c levels. **Conclusions:** While psychological distress is common among adults with diabetes, its direct impact on HbA1c may be attenuated in populations with strong religious and cultural coping practices. Conversely, metabolic factors, particularly total cholesterol and systolic blood pressure, alongside stress, showed stronger associations with HbA1c levels in this study population. These findings underscore the importance of integrating physiological, psychological, and culturally contextualised strategies in diabetes management to optimize outcomes.

## 1. Introduction

Recent studies in Saudi Arabia indicate that 30–40% of adults with type 2 diabetes experience moderate to severe psychological distress, including depression, anxiety, or stress, highlighting the need to evaluate these factors in relation to glycaemic control [1,2].

These emotional symptoms can arise from the ongoing psychological burden of managing a chronic condition that demands continuous self-care, regular monitoring, and lifestyle adjustments. Global research has shown that adults with diabetes are more likely to report depressive symptoms compared with the general population, and such symptoms have been linked to poorer adherence to treatment and self-management practices [3,4,5,6]. In diabetes care, depression does not exist in isolation: anxiety and stress frequently manifest alongside depression, creating overlapping emotional distress that may further influence health behaviours and metabolic outcomes. Studies exploring the biological and behavioural pathways connecting psychological distress and glycaemic control suggest multiple mechanisms through which emotional symptoms could relate to clinical outcomes. Stress responses activate neuroendocrine systems that increase cortisol levels, potentially leading to impaired insulin sensitivity and poorer glucose regulation [7,8,9]. Behaviourally, depression and anxiety were associated with reduced motivation, diminished engagement in physical activity, inconsistent glucose monitoring, and lower adherence to diet and medication, all of which can contribute to suboptimal glycaemic control [10]. Although most of these findings derive from international cohorts, they provide a foundational context for understanding how psychological distress could meaningfully intersect with metabolic control.

Within Saudi Arabia, research on psychological distress among adults with diabetes has grown, although few studies have directly examined its association with clinical measures such as haemoglobin A1c (HbA1c). Using the Depression Anxiety and Stress Scale-21 (DASS-21), Alzahrani and colleagues (2019) reported that significant proportions of type 2 diabetes patients attending primary healthcare centres experienced symptoms of depression, anxiety, and stress [2]. In this sample, individuals with higher HbA1c values were more likely to report elevated distress scores, indicating a potential association between poorer glycaemic control and psychological symptoms [2]. Similarly, a study conducted in the western region of Saudi Arabia found that higher DASS-21 depression and anxiety subscale scores were associated with lower quality of life and tended to cluster in patients with elevated HbA1c, although the research primarily examined quality-of-life outcomes rather than glycaemic control [11].

Beyond diabetes-specific studies, broader research in Saudi populations has documented elevated psychological distress among individuals with diabetes compared with those without the condition. For example, comparative investigations using general wellbeing measures found that patients with diabetes scored higher on depression and anxiety scales than non-diabetic groups, hinting at the emotional burden associated with chronic disease status [12]. While these studies do not directly link emotional distress with glycaemic markers, they support the premise that psychological symptoms were prevalent among people with diabetes in the local context.

International evidence also suggests associations between psychological distress measured by DASS-21 and metabolic control. In addition to general psychological distress, diabetes-specific distress has gained attention as an important psychological factor among individuals living with diabetes. Unlike depression and anxiety, diabetes distress reflects the emotional burden associated with disease management, including concerns related to treatment demands, complications, and self-care responsibilities. Research from diverse settings has found that higher depression and stress scores often correspond with increased HbA1c levels, underscoring the possibility that emotional distress and physiological control are interrelated [13,14,15]. However, differences in study designs, populations, and measurement tools have resulted in inconsistent findings across studies. Some research has found stronger links between diabetes-specific distress and self-management behaviours than between general depression and glycaemic outcomes, indicating that the emotional burden tied directly to diabetes may be more relevant than general mood symptoms in predicting metabolic control [16].

Although previous studies have reported a high prevalence of psychological distress among individuals with diabetes in Saudi Arabia, limited evidence exists regarding the simultaneous assessment of depression, anxiety, and stress in relation to objective glycaemic indicators such as HbA1c. Furthermore, most available studies have focused mainly on psychological symptoms, quality of life, or diabetes-related distress without integrating psychological, demographic, and biochemical factors within the same analytical model. Therefore, the current study aimed to examine the association between psychological distress measured by DASS-21 and glycaemic control among adults with diabetes mellitus in Saudi Arabia while considering relevant clinical and metabolic factors. This approach provides additional insight into the complex relationship between psychological wellbeing and metabolic control within the Saudi healthcare context.

## 2. Method

### 2.1. Study Design and Participants

This cross-sectional study was conducted between 21 January 2026 and 19 April 2026 across multiple healthcare settings to evaluate the association between psychological distress and glycaemic control among adults with diabetes mellitus in Saudi Arabia. A total of 546 adult patients required to have received a prior diabetes diagnosis from a physician were recruited from primary healthcare centres and outpatient clinics. A convenience sampling technique was employed for participant recruitment. Eligible patients attending the participating primary healthcare centres and outpatient clinics during the study period were invited to participate in the study. Patients who met the inclusion criteria and provided written informed consent were enrolled until the required sample size was achieved. Inclusion criteria included age ≥ 18 years, a confirmed diagnosis of diabetes, and willingness to participate. Also included were patients who were required to have received a prior diabetes diagnosis from a physician before enrolling. Patients with severe psychiatric disorders, cognitive impairment, or critical illness were excluded and those unable to provide informed consent or effectively communicate, including patients with severe mental illness, were excluded from participation. The sample included 289 males (52.9%) and 257 females (47.1%), with Saudi nationals comprising 93.4% of participants. Age distribution ranged from 18 to over 60 years, ensuring representation across adult age groups. Participant selection, data collection procedures, and handling of missing data were described according to STROBE recommendations for observational studies.

### 2.2. Sample Size Calculation

The sample size for the study was calculated using the formula n = Z^2^ × *p* (1 − *p*)/d^2^, where n represents the required sample size, Z is the standard normal variate corresponding to a 95% confidence level (1.96), *p* is the estimated prevalence, and d is the margin of error. Since the prevalence of psychological distress among patients with diabetes in the target population was not precisely known, a prevalence estimate of 50% was assumed to obtain the maximum sample size. Based on a 95% confidence level and a margin of error of 5%, the minimum required sample size was calculated to be 384 participants. However, to improve the precision of the findings, increase the statistical power of the study, and account for potential missing or incomplete data, participant recruitment continued throughout the study period. Consequently, a total of 546 eligible patients with diabetes mellitus were enrolled and included in the final analysis.

### 2.3. Data Collection

Prior to data collection, a study questionnaire was developed following an extensive review of the published literature and submitted to the Institutional Review Board (IRB) for ethical approval. The initial version of the questionnaire consisted of 21 items, which were critically reviewed and discussed by a panel comprising two consultant family medicine specialists. Following two rounds of discussion and refinement, a final questionnaire containing 15 items was approved and utilized for the study. Data collection was carried out by a team of well-trained researchers who conducted comprehensive health assessments at the hospital site using a standardized data collection protocol. During routine clinic visits, potential participants were approached and screened for eligibility. Patients diagnosed with diabetes mellitus were informed about the objectives and significance of the study, and their willingness to provide written informed consent and participate was sought. Written informed consent was obtained from all participants prior to enrollment. Once consent was provided, the research team collected the required demographic, clinical, and laboratory information. Cases with missing data for key variables were excluded from the final analysis. Future studies may use imputation methods to further reduce bias.

Additional clinical and laboratory data were retrieved from participants’ medical records available in the hospital database. These records provided information on age, glycated hemoglobin (HbA1c), total cholesterol, triglycerides, serum creatinine, urinary albumin levels, and the presence of other comorbid medical conditions. To assess obesity status, Body Mass Index (BMI) was calculated for each participant by dividing body weight in kilograms by the square of height in meters (kg/m^2^). Blood pressure measurements were obtained according to the recommendations of the World Health Organization/International Society of Hypertension. Three consecutive blood pressure readings were measured from the right arm using standardized mercury sphygmomanometers, and the average value was considered for analysis. This standardized approach was adopted to ensure consistency and accuracy of the measurements.

The data collection process was conducted by final-year MBBS students who received uniform training before the commencement of the study. This training ensured consistency in interviewing techniques, anthropometric measurements, and data recording procedures. Whenever missing information, discrepancies, or recording errors were identified, additional interactions with participants and verification from medical records were undertaken to ensure completeness and accuracy of the dataset. Furthermore, all measuring instruments were calibrated and standardized prior to use, thereby ensuring the collection of reliable and high-quality data in accordance with the scientific objectives of the study.

### 2.4. Assessment of Psychological Distress

Psychological distress was assessed using the validated Arabic and English versions of the DASS-21. The questionnaire was incorporated into the electronic data collection form and administered by trained researchers during the same visit when demographic and clinical information was collected. The scale provides scores for depression (normal 0–9, mild 10–12, moderate 13–20, severe 21–27, extremely severe 28–42) anxiety (normal 0–6, mild 7–9, Moderate 10–14, severe 15–19, extremely severe 20–42) and stress (normal 1–10, Mild 11–18, Moderate 19–26, severe 27–34, and extremely severe 35–42) (Supplementary Materials). Participants completed the DASS-21 in Arabic or English, according to preference, with assistance provided for those with literacy difficulties.

### 2.5. Glycaemic Control

Glycaemic control was evaluated using HbA1c levels, obtained from recent laboratory results. (The most recent HbA1c result recorded within the preceding three months was used for analysis.) Participants were categorised as having controlled diabetes (HbA1c < 7%) or uncontrolled diabetes (HbA1c ≥ 7%), consistent with American Diabetes Association guidelines.

### 2.6. Statistical Analysis

Data were analysed using SPSS version 26.0. Descriptive statistics were used to summarise demographic, clinical, and psychological characteristics. HbA1c was categorised into controlled (<7%) and uncontrolled (≥7%) groups for comparative analysis of psychological distress and clinical characteristics using chi-square tests. Before conducting multiple linear regression analysis, regression assumptions were assessed. Multicollinearity was evaluated using variance inflation factor (VIF) and tolerance values. Residual plots were examined to assess linearity, normality of residuals, and homoscedasticity. Influence diagnostics were also reviewed to identify potential influential observations. The model satisfied the assumptions required for linear regression analysis. In addition, HbA1c was analysed as a continuous outcome variable in multiple linear regression analysis to identify independent factors associated with variation in glycaemic levels. Variables with *p* < 0.2 were included as predictors in the final regression model. The regression model (using the Enter method) included psychological distress scores (stress, anxiety, and depression), BMI, systolic and diastolic blood pressure, total cholesterol, HDL, LDL, triglycerides, and urinary albuminuria as clinical predictors of HbA1c variation. Variables included in the regression model were selected according to the study objective, previous evidence regarding metabolic determinants of HbA1c, and availability of complete clinical measurements. Psychological distress variables and major metabolic parameters were included to evaluate their association with HbA1c. Other potential factors, including demographic characteristics, treatment-related variables, lifestyle behaviours, and socioeconomic factors, were considered possible residual confounders. Statistical significance was considered at *p* < 0.05.

### 2.7. Ethical Considerations

Participation was entirely voluntary, and written informed consent was obtained from all participants prior to enrollment. Participants were assured that all information would remain confidential and would be used solely for research purposes. They were informed of their right to withdraw from the study at any stage without consequences. This study was approved by the Research Ethics Committee (Institutional Review Board approval of research project No. # E-23-8197) of the Faculty of Medicine at KSU.

## 3. Results

### 3.1. Participant Characteristics

The current study included 546 adults diagnosed with diabetes mellitus. Of these, 52.9% were male (n = 289) and 47.1% were female (n = 257). The majority were Saudi nationals (n = 510, 93.4%), with a smaller proportion of non-Saudi participants (n = 36, 6.6%). Age distribution was relatively balanced: 35.3% were 18–40 years, 12.3% were 41–50 years, 25.5% were 51–60 years, and 26.9% were aged 61 years or older. Type 2 diabetes was predominant, affecting 76.9% of participants, while 23.1% had type 1 diabetes (Table 1). These demographics provide a representative view of adult diabetes patients in the sampled population.

### 3.2. Psychological Distress and Glycaemic Control

Psychological distress, measured using DASS-21, was evaluated in relation to glycaemic control (HbA1c). Among participants with controlled HbA1c (<7%), 55.0% had normal stress, 38.8% moderate, and 6.2% severe, whereas in the uncontrolled group (HbA1c ≥ 7%), these proportions were 47.7%, 41.6%, and 10.7%, respectively. Anxiety scores followed a similar pattern, with normal, moderate, and severe categories roughly evenly distributed across controlled and uncontrolled groups. Depression levels also showed no significant differences between glycaemic control groups. Chi-square analyses confirmed that stress (χ^2^ = 3.33, *p* = 0.18), anxiety (χ^2^ = 1.10, *p* = 0.57), and depression (χ^2^ = 3.19, *p* = 0.20) were not significantly associated with HbA1c outcomes (Table 2). These findings suggest that while psychological distress was prevalent, it did not directly differentiate between controlled and uncontrolled glycaemic states in this cohort.

### 3.3. Demographic and Biochemical Factors

In contrast, several demographic and metabolic parameters were significantly associated with glycaemic control. Nationality showed a strong relationship, with Saudi participants comprising 82.9% of the controlled group versus 96.6% of the uncontrolled group (χ^2^ = 29.3, *p* < 0.0001, 95% CI: 5.8–11.7). Lipid profile variables, including total cholesterol (χ^2^ = 69.7, *p* < 0.0001), HDL (χ^2^ = 4.0, *p* = 0.02), LDL (χ^2^ = 25.8, *p* < 0.0001), and triglycerides (χ^2^ = 4.56, *p* = 0.02), also showed significant associations with HbA1c (Table 3). Gender, BMI, and blood pressure measurements did not demonstrate significant relationships with glycaemic control.

### 3.4. Multiple Regression Analysis

A multiple linear regression model was conducted to evaluate the combined effect of psychological distress, BMI, blood pressure, and lipid parameters on HbA1c. The regression model included psychological distress scores, BMI, blood pressure measurements, lipid profile parameters, and urinary albuminuria to evaluate their association with HbA1c levels. Urine albuminuria showed a positive but non-significant association with HbA1c levels (B = 0.04, t = 1.379, *p* = 0.168). The model explained 14.8% of the variance in HbA1c (R^2^ = 0.148; adjusted R^2^ = 0.130), with a moderate predictive ability (R = 0.384). The model was statistically significant (F = 8.296, *p* < 0.001), with a standard error of 0.39211 (Table 4). Among predictors, stress was positively associated with HbA1c (B = 0.09, *p* = 0.015), indicating that higher stress levels corresponded with poorer HbA1c levels. Systolic blood pressure (B = 0.146, *p* = 0.006) and total cholesterol (B = 0.229, *p* < 0.001) were also significant contributors. Anxiety, depression, diastolic blood pressure, BMI, HDL, LDL, and triglycerides did not significantly predict HbA1c after controlling for other variables. These findings suggest that psychological distress components may have different relationships with HbA1c levels.

## 4. Discussion

In this study, psychological distress measured by the DASS-21 did not show a significant association with glycaemic control (HbA1c). The distribution of stress, anxiety, and depression scores was similar among participants with controlled and uncontrolled HbA1c. Specifically, although a slightly higher proportion of uncontrolled patients had severe stress compared with those with controlled glycaemia, the differences were not statistically significant. Anxiety and depression followed the same pattern. This finding contrasts with numerous international studies that report strong associations between psychological distress and poorer glycaemic outcomes [17,18,19,20,21]. These studies often suggest that elevated depressive symptoms contribute to lower adherence to self-care behaviours, resulting in higher HbA1c levels [22].

However, the absence of a significant association in the present study may be influenced by several factors, including sociocultural characteristics specific to the Saudi population. Previous studies have suggested that religious and cultural coping strategies may contribute to psychological resilience among Saudi individuals; however, these mechanisms were not directly assessed in the current study and should be considered as possible explanations requiring further investigation. Coping mechanisms in this population are deeply rooted in religious and cultural practices, which can buffer the impact of psychological distress on health behaviours. For example, Islamic religious coping including prayer, reliance on faith, and acceptance of life stressors as part of divine will has been identified as a primary strategy for managing stress among Saudis [23]. Previous research among Saudi adults with type 2 diabetes has suggested that illness perceptions, religious beliefs, and personal health control beliefs may influence self-care behaviours and diabetes management practices [24,25]. However, the direct role of religious coping in modifying the relationship between psychological distress and HbA1c requires further investigation [26,27,28]. Furthermore, cultural norms in Saudi Arabia may shape the expression and reporting of psychological symptoms. Some individuals may interpret emotional distress within a spiritual or social context rather than as discrete depressive or anxious pathology, which could dilute the strength of statistical associations with metabolic measures [2,29]. It is also possible that individuals with longstanding diabetes have adapted to their condition over time, relying more on structured religious routines and social networks that promote resilience, even in the presence of emotional symptoms.

The current study found that several demographic and biochemical variables were significantly associated with glycaemic control. Nationality, for instance, showed a strong relationship with HbA1c outcomes, with Saudi participants disproportionately represented in the uncontrolled group. This may reflect differences in lifestyle, dietary habits, or access to healthcare resources between Saudi and non-Saudi residents, consistent with prior regional studies highlighting sociocultural influences on diabetes management [2,30,31]. Biochemical markers, particularly lipid profile parameters, demonstrated significant associations with HbA1c levels. Elevated total cholesterol, LDL, and triglycerides were linked to higher HbA1c, while HDL cholesterol exhibited an inverse relationship. These findings align with established evidence that dyslipidaemia is closely intertwined with insulin resistance and poor glycaemic regulation in patients with type 2 diabetes [32,33,34]. The current results reinforce the need for integrated management strategies that target both glucose and lipid control to optimize metabolic outcomes.

Conversely, variables such as gender, BMI, and blood pressure did not show significant associations with HbA1c in this cohort, suggesting that within this population, metabolic control may be more strongly influenced by lipid abnormalities than by general anthropometric or hemodynamic measures. These findings emphasize the importance of individualized risk assessment and highlight potential targets for intervention, particularly in culturally specific populations where lifestyle patterns and genetic predispositions may modify traditional risk factor associations [35,36,37].

The multiple regression model in this current study demonstrated that stress, systolic blood pressure, and total cholesterol were significant predictors of HbA1c levels, collectively explaining 14.8% of the variance. Stress was positively associated with HbA1c levels (B = 0.09, *p* = 0.015), suggesting that higher perceived stress was associated with a modest increase in HbA1c levels. Stress may influence glycaemic control through behavioural and physiological pathways. Behaviourally, stress can increase hedonic food intake and reduce adherence to self-care, while physiologically, stress-induced cortisol elevation may impair insulin sensitivity, contributing to higher HbA1c. Similarly, systolic blood pressure (B = 0.146, *p* = 0.006) and total cholesterol (B = 0.229, *p* < 0.001) showed significant positive relationships with HbA1c levels, highlighting the important role of metabolic and cardiovascular parameters in diabetes management. Other factors, including anxiety, depression, BMI, HDL, LDL, diastolic blood pressure, and triglycerides, were not significant predictors after adjusting for covariates, reinforcing the notion that not all psychological or anthropometric factors directly impact glycaemic outcomes. These findings suggest that psychological distress components may not be associated with HbA1c equally. While anxiety and depression were not significant predictors after adjustment, stress showed a modest but significant association with HbA1c, indicating that stress-related pathways may contribute to glycaemic variation among adults with diabetes. Previous research has shown that religious coping strategies, such as prayer and faith-based cognitive framing, can buffer the effects of stress and reduce its behavioural consequences [23,24,25]. Older participants, in particular, may rely on these cultural and spiritual resources, mitigating the potential negative effect of anxiety and depression on metabolic control. In line with regional studies, this emphasizes that in populations with strong cultural and religious frameworks, psychological distress may be present but its direct influence on glycaemic regulation can be attenuated [2]. The regression results indicate the multifactorial nature of HbA1c variation, where stress was associated with metabolic factors including systolic blood pressure and cholesterol levels. Clinically, these findings highlight the need to integrate both physiological and culturally contextualised psychosocial strategies in diabetes care to optimize outcomes. Variations in study populations, diabetes type distribution, disease duration, healthcare settings, and psychological assessment instruments may influence the observed relationship between psychological distress and glycaemic control. Furthermore, behavioural and clinical factors, including medication adherence, lifestyle behaviours, and diabetes self-management practices, may modify this relationship. Therefore, the association between psychological distress and HbA1c should be interpreted within the broader clinical and social context. Although the regression model was statistically significant, it explained 14.8% of the variation in HbA1c, indicating that a considerable proportion of glycaemic variability remains influenced by other factors not assessed in this study. Therefore, the identified predictors should be interpreted as contributing factors rather than complete determinants of glycaemic control. Future studies incorporating treatment-related, behavioural, and socioeconomic variables may provide a more comprehensive understanding of factors influencing HbA1c.

### Limitations

This study has a few limitations that should be considered. First, its cross-sectional design prevents determination of causal relationships between psychological distress and glycaemic control. Second, convenience sampling from healthcare centers in Riyadh may limit the generalisability of findings to other regions or populations; the findings should be interpreted considering possible selection bias and residual confounding. Third, psychological distress was measured using self-reported DASS-21, which may be influenced by reporting bias or cultural factors that affect disclosure of emotional symptoms. Fourth, several important clinical and behavioural factors were not included in the analysis, including diabetes duration, treatment regimen, medication adherence, physical activity, dietary habits, smoking status, socioeconomic status, and diabetes-related complications. These factors showed associations with HbA1c levels and could contribute to additional variation in HbA1c levels. Finally, the use of single-point laboratory measurements means temporal fluctuations in HbA1c and biochemical parameters were not captured. Despite these limitations, the study provides valuable insight into the relationship between psychological distress, metabolic parameters, and cultural coping practices in adults with diabetes in Saudi Arabia, guiding future research and clinical interventions. Clinically, these findings suggest that interventions targeting stress management, cardiovascular risk optimization, and culturally sensitive psychosocial support may help improve diabetes management among adults in Saudi Arabia. Screening for stress and providing structured educational programs may enhance self-care and metabolic control.

## 5. Conclusions and Recommendations

The current study demonstrates that psychological distress is common among adults with diabetes in Saudi Arabia. However, among the assessed psychological factors, only stress showed a significant association with HbA1c, whereas anxiety and depression were not independent predictors after adjustment for clinical and biochemical factors. Instead, metabolic factors, particularly total cholesterol and systolic blood pressure, alongside stress, showed stronger associations with HbA1c levels in this study population. The findings suggest that cultural and religious factors may represent important contextual influences on the relationship between psychological distress and diabetes management. However, these factors were not directly measured in this study and require further investigation.

Clinically, these results emphasize the need for integrated diabetes care strategies that address both metabolic control and psychosocial support. Screening for stress, optimizing cardiovascular risk factors, and encouraging culturally sensitive coping interventions such as faith-based support or community engagement may support better diabetes management and should be further evaluated. Future research should explore longitudinal effects, incorporate additional behavioural and lifestyle variables, and expand to diverse regions to strengthen evidence-based interventions tailored to the Saudi population.

## Figures and Tables

**Table 1:** Demographic and Clinical Characteristics of the Study Population.

Item	Categories	n (%)
Gender	Male	289 (52.9)
	Female	257 (47.1)
Nationality	Non-Saudi	36 (6.6)
	Saudi	510 (93.4)
Age	18–40	193 (35.3)
	41–50	67 (12.3)
	51–60	139 (25.5)
	61-more	147 (26.9)
Weight	KG	546 (100)
Height	CM	525 (96.6)
Diabetes type	Type 1	126 (23.1)
	Type 2	420 (76.9)
HbA1C		540 (98.9)
Systolic blood pressure	mmHg	546 (100)
Diastolic blood pressure	mmHg	546 (100)
Total cholesterol	mmol/L	542 (99.3)
LDL	mmol/L	542 (99.3)
HDL	mmol/L	542 (99.3)
Triglycerides	mmol/L	536 (98.2)
serum creatinine	mmol/L	536 (98.2)
Urinary albumin/creatinine ratio	mmol/L	546 (100)
Depression		546 (100)
Anxiety		546 (100)
Stress		546 (100)

**Table 2:** Psychological Distress Scores in Controlled and Uncontrolled Diabetes.

			HbA1c < 7% = Controlled			HbA1c ≥ 7% = Uncontrolled		
Item	Categories	n (%)	n (%)	95% CI	*p*-Value	n (%)	95% CI	*p*-Value
Stress	Normal	270 (49.5)	71 (55.0)	|		196 (47.7)	|	
	Moderate	224 (41.0)	50 (38.8)	0.84 (0.56–1.27)	0.42	171 (41.6)	1.05 (0.80–1.3)	0.71
	Severe	52 (9.5)	8 (6.2)	0.58 (0.26–1.28)	0.18	44 (10.7)	1.16 (0.74–1.8)	0.49
Anxiety	Normal	231 (42.3)	55 (42.6)	|		173 (42.1)	|	
	Moderate	224 (41.0)	56 (43.4)	1.05 (0.69–1.59)	0.81	165 (40.1)	0.98 (0.74–1.3)	0.9
	Severe	91 (16.7)	18 (14.0)	0.83 (0.46–1.49)	0.53	73 (17.8)	1.07 (0.74–1.5)	0.71
Depression	Normal	233 (42.7)	55 (42.6)	|		173 (42.1)	|	
	Moderate	187 (34.2)	56 (43.4)	1.26 (0.83–1.92)	0.26	165 (40.1)	1.18 (0.89–1.58)	0.23
	Severe	105 (96.2)	18 (14.0)	0.72 (0.40–1.29)	0.27	73 (17.8)	0.93 (0.65–1.33)	0.71

Note: | indicates the reference category used for the calculation of the odds ratio (OR).

**Table 3:** Association Between Psychological Distress, Demographic Characteristics, Clinical Factors, and Glycaemic Control Among Adults with Diabetes Mellitus.

Item	Categories	Controlled HbA1c < 7% n (%)	HbA1c ≥ 7% = Uncontrolled	χ^2^ (*p*-Value)	95% CI
Stress	Normal	71 (55.0)	196 (47.7)	3.33 (0.18)	
	Moderate	50 (38.8)	171 (41.6)		
	Severe	8 (6.2)	44 (10.7)		
Anxiety	Normal	55 (42.6)	173 (42.1)	1.10 (0.57)	
	Moderate	56 (43.4)	165 (40.1)		
	Severe	18 (14.0)	73 (17.8)		
Depression	Normal	61 (47.3)	170 (41.4)	3.19 (0.20)	
	Moderate	36 (27.9)	148 (36.0)		
	Severe	28 (21.7)	76 (18.5)		
Nationality	Non-Saudi	22 (17.1)	14 (3.4)	29.3 (<0.0001)	5.8 (2.8–11.7)
	Saudi	107 (82.9)	397 (96.6)		
Gender	Male	73 (56.6)	210 (51.1)	1.18 (0.16)	0.80 (0.53–1.1)
	Female	56 (43.4)	201 (48.9)		
BMI	Normal < 25	34 (26.4)	92 (22.4)	5.05 (0.08)	
	Overweight ≥ 25 to <30	26 (20.2)	133 (32.4)		
	Obese ≥ 30 or more	55 (42.6)	179 (43.6)		
Systolic blood pressure (mmHg)	Normal ≤ 120	33 (25.6)	81 (19.7)	2.03 (0.09)	1.4 (0.88–2.2)
	Not Normal > 120	96 (74.4)	330 (80.3)		
Diastolic blood pressure (mmHg)	Normal ≤ 80	84 (65.1)	292 (71.0)	1.63 (0.12)	0.76 (0.50–1.1)
	Not Normal > 80	45 (34.9)	119 (29.0)		
Total cholesterol (mmol/L)	Normal < 5.2	123 (95.3)	224 (54.5)	69.7 (<0.0001)	16.7 (7.2–38.8)
	Not Normal ≥ 5.2	6 (4.7)	183 (44.5)		
HDL (mmol/L)	Normal > 1	121 (93.8)	356 (86.6)	4.0 (0.02)	2.16 (1.0–4.69)
	Not Normal ≤ 1.0	8 (6.2)	51 (12.4)		
LDL (mmol/L)	Normal < 2.6	87 (67.4)	170 (41.4)	25.8 (<0.0001)	2.88 (1.9–4.38)
	Not Normal ≥ 2.6	42 (32.6)	237 (57.7)		
Triglycerides (mmol/L)	Normal < 1.7	102 (79.1)	278 (67.6)	4.56 (0.02)	1.67 (1.04–2.6)
	Not normal ≥ 1.7	27 (20.9)	123 (29.9)		

**Table 4:** Multiple Linear Regression Analysis of Factors Associated with HbA1c Levels Among Adults with Diabetes Mellitus. (**A**) Multiple Linear Regression Analysis of Factors Associated with Glycemic Control (HbA1c) Among Diabetic Patients. (**B**) Model summary for predictors of HbA1c levels.

**A**						
**Variables**	**B**	**SE**	**Standardized β**	**t-Value**	***p*** **-Value**	**95% CI**
Stress score	0.09	0.037	0.136	2.443	0.015 *	0.018–0.162
Anxiety score	−0.05	0.035	−0.084	−1.412	0.159	−0.119–0.019
Depression score	−0.01	0.029	−0.017	−0.337	0.736	−0.066–0.046
BMI	−0.037	0.026	−0.071	−1.392	0.164	−0.088–0.015
Urine albuminuria	0.04	0.029	0.016	1.379	0.168	0.018–0.071
Systolic BP	0.146	0.053	0.142	2.755	0.006 *	0.042–0.251
Diastolic BP	−0.031	0.042	−0.034	−0.736	0.462	−0.114–0.052
Total Cholesterol	0.229	0.05	0.26	4.551	<0.001 *	0.130–0.328
HDL cholesterol	0.059	0.062	0.044	0.957	0.339	−0.062–0.180
LDL cholesterol	0.08	0.047	0.095	1.713	0.087	−0.012–0.172
Triglycerides	0.051	0.04	0.055	1.255	0.21	−0.029–0.130
**B**						
**Model Statistics**				**Value**		
R				0.384		
R^2^				0.148		
Adjusted R^2^				0.13		
F-statistic				8.296		
*p*-value				<0.001		

Dependent variable: HbA1c (%). Predictors included stress, anxiety, depression, BMI, systolic blood pressure, diastolic blood pressure, total cholesterol, HDL cholesterol, LDL cholesterol, triglycerides, and urinary albuminuria. * significant value.

## Data Availability

The data presented in this study are available on request from the corresponding author. The data are not publicly available due to ethical and privacy restrictions.

## Supplementary Materials

The following supporting information can be downloaded at: https://www.mdpi.com/article/10.3390/healthcare14182978/s1.
