# Supplementary material for: Association Between Psychological Distress and Glycaemic Control Among Adults with Diabetes Mellitus in Saudi Arabia
Source: Healthcare (Basel). 2026 Sep 11;14(18):2978. doi: 10.3390/healthcare14182978 (PMC13606292; doi:10.3390/healthcare14182978)
Supplement: Supplementary file 1 [file healthcare-14-02978-s001.zip › healthcare-4422332-supplementary.pdf]

Supplementary Materials

Study Population:

546 adults with diabetes mellitus
↓

Assessment:

- DASS-21:
  - Stress;
  - Anxiety;
  - Depression.
- Clinical variables:
  - HbA1c;
  - Blood pressure;
  - Lipid profile;
  - BMI.

↓

Analysis:

- Chi-square: Controlled vs uncontrolled HbA1c;
- Multiple linear regression: Predictors of HbA1c.

↓

Main Findings:

✓ Stress, anxiety, and depression were not significantly different between HbA1c groups;
✓ Stress showed modest association with continuous HbA1c;
✓ Total cholesterol and systolic blood pressure were significant metabolic predictors.
